# Effect of PCSK9 inhibitor on early neurological deterioration in acute ischemic stroke patients with a history of coronary heart disease: a study protocol for a randomized controlled trial in Dalian, China

**DOI:** 10.1186/s13063-024-08709-2

**Published:** 2025-01-06

**Authors:** Xing Gong, Xinting Yu, Lanlan Pu, Yang Jiao, Lin Liu, Hua Cao, Xiaofei Ji

**Affiliations:** 1 Department of Neurology, the First Affiliated Hospital of Dalian Medical University, Dalian, Liaoning Province China; 2 Dalian Innovation Institute of Stem Cell and Precision Medicine, Dalian, People’s Republic of China

**Keywords:** Early neurological deterioration, Acute ischemic stroke, Coronary heart disease, PCSK9 inhibitors

## Abstract

**Background:**

Early neurological deterioration (END) is a critical determinant influencing the short-term prognosis of acute ischemic stroke (AIS) patients and is associated with increased mortality rates among hospitalized individuals. AIS frequently coexists with coronary heart disease (CHD), complicating treatment and leading to more severe symptoms and worse outcomes. Shared risk factors between CHD and AIS, especially elevated low-density lipoprotein cholesterol (LDL-C), contribute to atherosclerosis and inflammation, which worsen brain tissue damage. Proprotein convertase subtilisin/kexin type 9 (PCSK9) inhibitors offer a promising treatment option. They effectively lower LDL-C levels and may help reduce END in AIS patients with CHD. This study aims to evaluate how effective PCSK9 inhibitors are in reducing END among this high-risk group and to provide new insights for treatment strategies.

**Methods:**

This is a prospective, randomized, parallel-group, blinded-endpoint, single-center clinical study. A total of 156 AIS patients with a history of CHD and within 24 h from symptom onset will be recruited and randomized in a 1:1 allocation to either the PCSK9 inhibitor combined with statin treatment group (PI group) or the statin monotherapy group (AT group). The PI group will receive a combination therapy consisting of evolocumab and rosuvastatin calcium tablets, while the AT group will receive only oral rosuvastatin calcium tablets. The trial duration will last for 90 days and comprise three phases: screening, treatment intervention, and follow-up assessments. Participants will undergo comprehensive examinations and assessments on days 1, 7, 30, and 90 after enrollment.

**Discussion:**

This study aims to investigate the potential preventive effects of PCSK9 inhibitors on END in AIS patients with a history of CHD. A positive outcome from this trial could provide novel clinical strategies for reducing the incidence of END and improving the short-term prognosis among these stroke patients.

**Trial registration:**

China Clinical Trial Registry, ChiCTR2300078198. Registered on 30 November 2023.

**Supplementary Information:**

The online version contains supplementary material available at 10.1186/s13063-024-08709-2.

## Administrative information

**Table:** 

Title {1}	Effect of PCSK9 inhibitor on early neurological deterioration in acute ischemic stroke patients with a history of coronary heart disease: a study protocol for a randomized controlled trial in Dalian, China
Trial registration {2a and 2b}	Protocol Record number is YJ-KY-2024–109. WHO Trial Registration DataSet is not available. Trial was registered at www. chictr.org
Protocol version {3}	Version1.1, 30 November 2023 with the identifier of ChiCTR2300078198
Funding {4}	This study is supported by National Natural Science Youth Fund Project (Grant No.82301549), Liaoning Provincial Department of Education Research Project (No.LJKMZ20221283) and Liaoning Provincial Natural Science Foundation Project (No.2024-BS-167)
Author details {5a}	^1^Department of Neurology, the First Affiliated Hospital of Dalian Medical University, Dalian, Liaoning Province, China. ^2^ Dalian Innovation Institute of Stem Cell and Precision Medicine, Dalian, People’s Republic of China;
Name and contact information for the trial sponsor {5b}	National Natural Science Foundation of China, 83 Shuangqing Road, Haidian District, Beijing, China. Phone: + 86 010–62328591 Liaoning Provincial Department of Education, 169 Youth Street, Heping District, Shenyang City, Liaoning Province, China. Phone: + 86 024–22822222 Liaoning Provincial Department of Science and Technology, 6 Jianshe Avenue, Heping District, Shenyang City, Liaoning Province, China. Phone: + 86 024–22820222
Role of sponsor {5c}	The sponsors had no role in the study design, data collection, analysis, interpretation, or manuscript writing

## Introduction

### Background and rationale {6a}

Early neurological deterioration (END) is defined as the progressive or stepwise exacerbation of neurological deficits within 7 days after onset of stroke, with an incidence ranging from 5 to 40% [1–4]. END is a crucial factor in determining short-term prognosis in patients with acute ischemic stroke (AIS); furthermore, it is associated with increased mortality rates among hospitalized individuals [5]. Given the significant clinical implications of END, preventing its occurrence is of paramount importance.

AIS and coronary heart disease (CHD) are leading causes of death worldwide, significantly impacting patients’ quality of life and prognosis while also placing a substantial burden on healthcare systems [6, 7]. Notably, in clinical practice, AIS often coexists with CHD, leading to more severe symptoms, higher mortality risks, and poorer prognoses compared to patients without CHD [8, 9]. However, the best treatment plan for these patients is still uncertain.

CHD and large artery atherosclerotic stroke share common risk factors. Elevated low-density lipoprotein cholesterol (LDL-C) leads to atherosclerosis [3]. In AIS, high levels of oxidized low-density lipoprotein (ox-LDL) activate pro-inflammatory signaling pathways in the affected area. This activation triggers the release of pro-inflammatory cytokines, including interleukin 6 (IL-6), interleukin 8 (IL-8), and tumor necrosis factor alpha (TNF-α) [10]. These cytokines worsen brain tissue damage in the ischemic penumbra [11]. Ox-LDL activation also raises the levels of matrix metalloproteinases (MMPs), which promote plaque instability [12, 13]. Detachment of unstable plaques and local thrombus accumulation that block distal blood vessels are key mechanisms of END in AIS [14]. According to the “Shanghai Expert Consensus on Enhanced Blood Lipid Management in Ischemic Stroke,” achieving LDL-C targets in AIS patients can reduce the risk of secondary early neurological deterioration (END) events and other vascular recurrences Tables 1 and 2.

**Table 1:** Inclusion and exclusion criteria

Inclusion Criteria	1.Age between 18 and 80 years. 2.Acute ischemic stroke patients within 24 hours of onset. 3.Documented history of atherosclerotic coronary artery disease. 4.First-time onset or recurrent patients without residual sequelae such as limb paralysis affecting the current NIHSS score; previous mRS score ≤1. 5.Voluntary signing of informed consent.
Exclusion Criteria	1.Hemorrhagic or mixed-type stroke. 2.Patients with cerebral arteriovenous malformations or intracranial tumors. 3.Inability to control severe hypertension (systolic blood pressure persistently ≥180 mmHg or diastolic blood pressure ≥110 mmHg after active treatment), severe infections (meeting criteria such as elevated body temperature ≥38°C, signs of shock, infection-related consciousness impairment, respiratory failure, and blood gas analysis PO_2_ <60 mmH_2_O), or abnormal liver function (ALT > 100 IU/L or AST > 80 IU/L), renal dysfunction (glomerular filtration rate < 30 ml/min), or patients with bleeding tendencies in the blood system (platelets < 60×10^9^/L or APTT > 60 seconds or INR > 3). 4.History of previous cerebral hemorrhage. 5.Occurrence of retinal or visceral bleeding within 30 days. 6.Use of PCSK9 inhibitors in the three months preceding the onset. 7.Patients with impaired consciousness. 8.Known allergy or intolerance to PCSK9 inhibitors or statin components. 9.Pregnant or lactating women and those planning pregnancy within six months. 10.Participants deemed unsuitable for the trial by the investigator.

*NIHSS* National Institutes of Health Stroke Scale, *mRS* Modified Rankin Scale, *ALT* Alanine Aminotransferase, *AST* Aspartate Aminotransferase, *APTT* Activated Partial Thromboplastin Time, *INR* International Normalized Ratio

**Table 2:** Trial registration data

Data category	Information
Primary registry and trial identifying number	China Clinical Trial Registry ChiCTR2300078198
Date of registration in primary registry	30 November, 2023
Public title	PCSK9 Inhibitor Prevents Early Neurological Deterioration in Acute Ischemic Stroke Patients with a History of Coronary Heart Disease: A Clinical Trail
Scientific title	PCSK9 Inhibitor Prevents Early Neurological Deterioration in Acute Ischemic Stroke Patients with a History of Coronary Heart Disease: A Clinical Trail
Study leader	Xiaofei Ji
Primary sponsor	First Affiliated Hospital of Dalian Medical University
Secondary sponsor	First Affiliated Hospital of Dalian Medical University
Source(s) of funding	None
Study type	Interventional study Allocation: randomized intervention model
Countries of recruitment	China
Intervention(s)	PI group: Subcutaneous injection of evolocumab 140mg every two weeks, combined with oral administration of rosuvastatin 20mg once nightly.
	AT group: oral administration of rosuvastatin 20mg once nightly
Key inclusion and exclusion criteria	Ages eligible for study: between 18 and 80 years Sexes eligible for study: both Accepts healthy volunteers: no
	Inclusion criteria: Acute ischemic stroke patients with a History of Coronary Heart Disease within 24 hours of onset
	Exclusion criteria: Hemorrhagic or mixed-type stroke. Cerebral arteriovenous malformations or intracranial tumors. Uncontrolled severe hypertension. History of previous cerebral hemorrhage. Retinal or visceral bleeding within 30 days. Use of PCSK9 inhibitors in the past three months. Impaired consciousness. Known allergy or intolerance to PCSK9 inhibitors or statin components. Pregnancy, lactation, or planning pregnancy within six months. Deemed unsuitable for the trial by the investigator.
Date of first enrolment	1 December, 2023
Target sample size	156
Recruitment status	Recruiting
Primary outcome(s)	END and severe END
Key secondary outcomes	Rate of good prognosis at 30 and 90 days. Recurrence rate of symptomatic cardiovascular and cerebrovascular events within 90 days. Changes in blood biochemical indices and inflammatory factors.

In this context, proprotein convertase subtilisin/kexin type 9 (PCSK9) inhibitors, a novel and potent class of lipid-lowering drugs, are gaining significant recognition. By competitively inhibiting the binding of PCSK9 to low-density lipoprotein receptors (LDLR), they reduce LDLR degradation effectively [15], thus lowering circulating low-density lipoprotein cholesterol (LDL-C) levels. Studies have demonstrated that PCSK9 inhibitors can suppress pro-inflammatory cytokines release [10], reduce endothelial cell apoptosis [16], and inhibit platelet activation [17]. As a result, these inhibitors hold promise in preventing atherosclerosis progression and reducing cardiovascular events. This study aims to assess the effectiveness of PCSK9 inhibitors in lowering the incidence of END among AIS patients with a history of CHD.

The findings of this study will not only elucidate the potential role of PCSK9 inhibitors in AIS patients with a history of CHD but also provide novel insights and directions for the treatment of this specific patient population.

### Objectives {7}

The primary objective of this study is to investigate the effects of PCSK9 inhibitors on END in AIS patients with a history of CHD within 24 h of symptom onset. Secondary objectives include evaluating changes in daily living abilities, recurrence rates of cardiovascular and cerebrovascular events within 90 days, alterations in blood biochemical indices/markers, and variations in inflammatory factors between the two patient groups after treatment.

### Trial design {8}

**Fig. 1:**
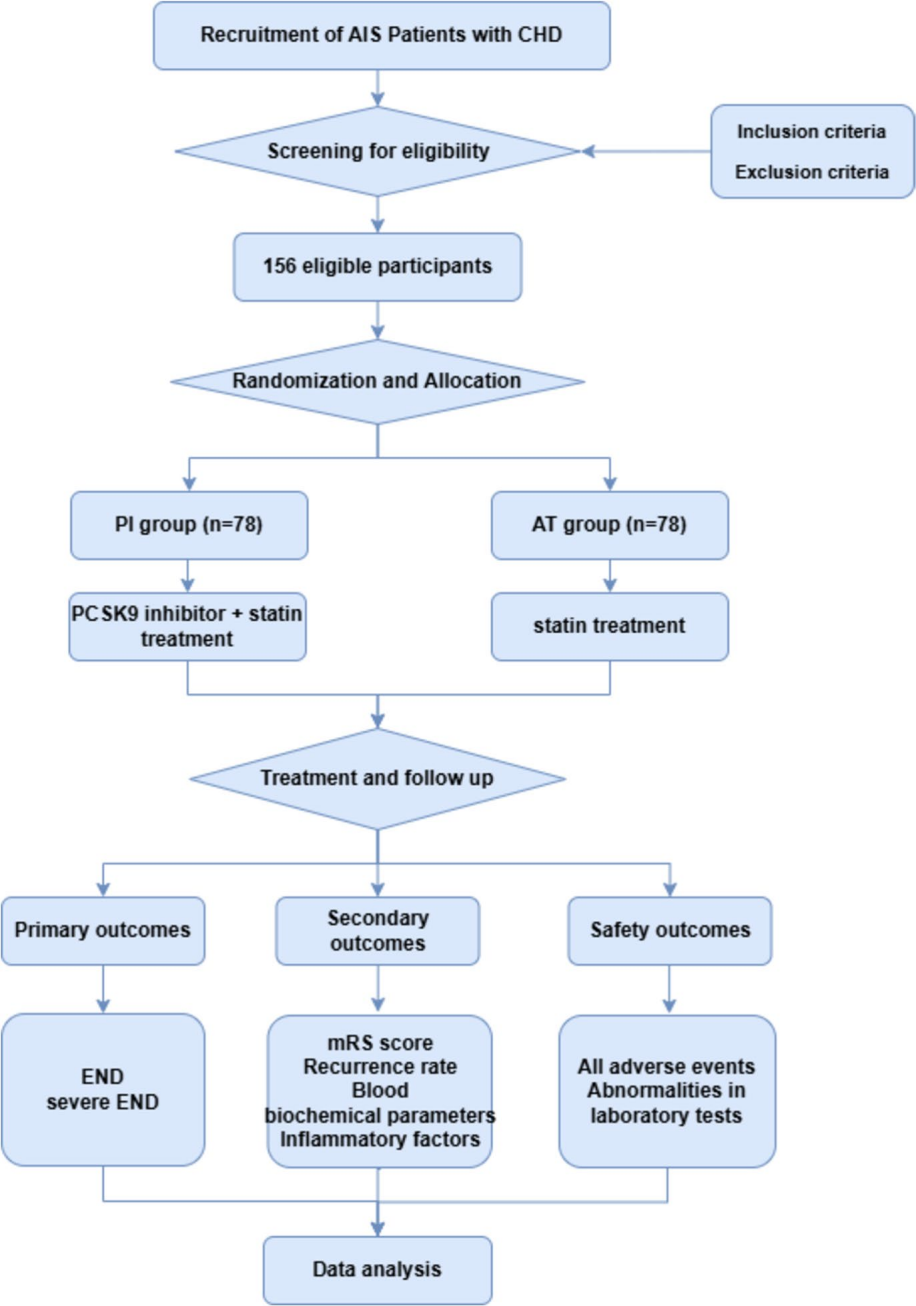
Expected trial plan. Not included in the diagram are participants who are excluded or stop participation at any time for various reasons

This is a prospective, randomized, parallel-group, blinded-endpoint, single-center clinical study aimed at investigating the preventive effects of PCSK9 inhibitors on END in patients with AIS and a history of CHD within 24 h of symptom onset. The study will be conducted from December 2023 to November 2025 at the Neurology Department of the First Affiliated Hospital of Dalian Medical University. A total of 156 AIS patients with a history of CHD and symptom onset within 24 h will be recruited and randomly assigned to either the PCSK9 inhibitor combined with statin treatment group (PI group) or the statin monotherapy group (AT group). The PI group will receive a combination therapy consisting of evolocumab and rosuvastatin calcium tablets, while the AT group will receive only oral rosuvastatin calcium tablets alone. The trial duration will be 90 days and consist of three phases: screening, treatment, and follow-up. Participants will undergo examinations and assessments on days 1, 7, 30, and 90 after enrollment. Written informed consent is mandatory for all participants prior to trial entry. The flow chart of the trial is illustrated in Fig. 1.

## Methods: participants, interventions, and outcomes

### Study setting {9}

The study will be conducted at the First Affiliated Hospital of Dalian Medical University, located in Dalian, China. This hospital will serve as the primary site for the recruitment of participants and the implementation of the clinical trial. The anticipated duration of the study is 2 years, encompassing participant enrollment, intervention administration, assessments, and data analysis.

### Eligibility criteria {10}

Participants will be recruited from the Neurology Department of the First Affiliated Hospital of Dalian Medical University based on predefined inclusion criteria. Only eligible individuals will be invited to enroll in the study.

The inclusion criteria are as follows:

1. Age between 18 and 80 years
2. Acute ischemic stroke patients within 24 h of onset
3. Documented history of atherosclerotic coronary artery disease
4. First-time onset or recurrent patients without residual sequelae such as limb paralysis affecting the current NIHSS score; previous mRS score ≤ 1
5. Voluntary signing of informed consent

The exclusion criteria are as follows:

1. Hemorrhagic or mixed-type stroke
2. Patients with cerebral arteriovenous malformations or intracranial tumors
3. Inability to control severe hypertension (systolic blood pressure persistently ≥ 180 mmHg or diastolic blood pressure ≥ 110 mmHg after active treatment), severe infections (meeting criteria such as elevated body temperature ≥ 38 °C, signs of shock, infection-related consciousness impairment, respiratory failure, and blood gas analysis PO_2_ < 60 mmH_2_O), or abnormal liver function (ALT > 100 IU/L or AST > 80 IU/L), renal dysfunction (glomerular filtration rate < 30 ml/min), or patients with bleeding tendencies in the blood system (platelets < 60 × 10.^9^/L or APTT > 60 s or INR > 3)
4. History of previous cerebral hemorrhage
5. Occurrence of retinal or visceral bleeding within 30 days
6. Use of PCSK9 inhibitors in the three months preceding the onset
7. Patients with impaired consciousness
8. Known allergy or intolerance to PCSK9 inhibitors or statin components
9. Pregnant or lactating women and those planning pregnancy within 6 months
10. Participants deemed unsuitable for the trial by the investigator

### Who will take informed consent? {26a}

All eligible participants will be invited to participate in this study. Two trained research personnel will conduct face-to-face sessions with potential participants or their authorized surrogates, wherein they will provide comprehensive explanations regarding the study’s objectives, procedures, associated risks, and the rights of participants. They will address any queries from participants or surrogates and ensure a thorough comprehension of the provided information. Prior to enrollment, informed consent will be obtained through signed written consent forms from either the participant or their authorized surrogate.

### Additional consent provisions for collection and use of participant data and biological specimens {26b}

The consent form requests participants’ permission for the continued use of their data even in the event of trial withdrawal. Additionally, participants are requested to grant permission to the research team for sharing pertinent data with applicable regulatory authorities. It is noteworthy that the trial does not encompass the collection of biological specimens.

## Interventions

### Explanation for the choice of comparators {6b}

The selection of comparators in this study is based on the current standard treatment for AIS. Intensified lipid-lowering therapy with statins represents the established treatment regimen for AIS patients with a history of coronary heart disease (CHD). Therefore, comparing PCSK9 inhibitor combined with statin treatment to statin monotherapy aims to evaluate the additional benefits of PCSK9 inhibitors in preventing END in AIS patients with a history of CHD.

### Intervention description {11a}

After signing the informed consent, participants will be randomly allocated to either the PI group (intervention group) or the AT group (control group). Participants in the PI group will receive a subcutaneous injection of 140 mg (1 ml) of evolocumab (Rephatha®, Amgen Inc.) and orally take 20 mg of rosuvastatin (Crestor®, AstraZeneca Inc.) calcium tablets within 1 h of randomization. Starting from the following day, they will receive 20 mg of rosuvastatin calcium tablets orally every evening and 140 mg of evolocumab subcutaneously once every 2 weeks. Participants in the AT group will receive 20 mg of rosuvastatin calcium tablets orally within 1 h of randomization. Starting from the next day, they will continue to orally receive 20 mg of rosuvastatin calcium tablets orally every evening.

### Criteria for discontinuing or modifying allocated interventions {11b}

Participants are allowed to withdraw from the study at any time due to various reasons, including failure to meet the inclusion criteria, inability to continue with the intervention treatment, or participant request.

### Strategies to improve adherence to interventions {11c}

To minimize the risk of missing data, each participant will be furnished with paper reminders containing precise dates and times for all scheduled sessions. Additionally, the investigator will conduct weekly phone calls to prompt participants about upcoming sessions and inquire about their adherence to medication.

### Relevant concomitant care permitted or prohibited during the trial {11d}

During the trial, there will be no regulations or restrictions placed on concomitant care or interventions.

### Provisions for post-trial care {30}

N/A. Given the low-risk nature of this study, we do not anticipate the necessity for posttrial care provisions.

### Outcomes {12}

The primary outcome of this study will be the END within 7 days. END will be defined as a 2-point increase in the NIHSS score or a 1-point increase in motor function (specifically NIHSS Items 5, 6, and 7) within 24 h to 7 days of AIS onset. To address the potential impact of END severity on the reliability of the primary outcome, we will conduct a sensitivity analysis using a higher threshold: a 4-point worsening in the NIHSS or a 2-point increase in motor function, which will be classified as severe END.

The secondary outcomes of the study will include:

1. Evaluation the rate of good prognosis at 30 and 90 days. Good prognosis will be defined as a modified Rankin Scale (mRS) score of 2 or lower, which indicates either no disability or minor disability
2. Assessment of the recurrence rate of symptomatic cardiovascular and cerebrovascular events within 90 days
3. Analysis of changes in blood biochemical indices (including liver function, renal function, lipid profile, etc.) before and after treatment, measured at baseline, 7 days, 30 days, and 90 days
4. Examination of changes in inflammatory factors (interleukin-6, IL-6; interleukin-8, IL-8; tumor necrosis factor-alpha, TNF-α) before and after treatment, assessed at baseline and 7 days

Safety outcomes will be documented by recording the types and frequencies of all adverse events and abnormalities in laboratory tests throughout the study duration. Safety evaluations will include assessments of the patients’ general condition, vital signs, blood routine tests, blood biochemistry, liver and kidney function, coagulation function, and neuroimaging examinations. All participants will undergo assessments before treatment initiation and at the initial treatment milestones of 30 days and 90 days.

### Participant timeline {13}

**Fig. 2:**
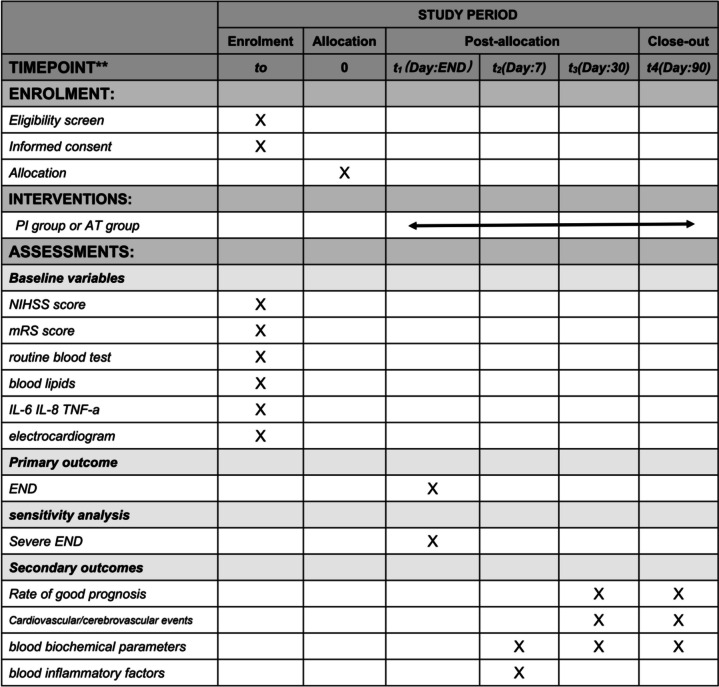
Standard Protocol Items: Recommendation for Interventional Trials (SPIRIT). NIHSS, National Institutes of Health Stroke Scale; mRS, modified Rankin Scale; IL-6, interleukin-6; IL-8, interleukin-8; TNF-α, tumor necrosis factor-alpha

The standard protocol is depicted in Fig. 2.

### Sample size {14}

This study is a randomized controlled trial with the intervention group as the PI group and the control group as the AT group. The study involves AIS patients with a confirmed history of CHD, and the primary outcome is the END in the two groups.

Based on our retrospective observation of 21 AIS patients with a history of CHD and no prior use of PCSK9 inhibitors, hospitalized at the First Affiliated Hospital of Dalian Medical University from April 2022 to June 2023, a total of 7 patients exhibited END within 24 h to 7 days of disease onset, accounting for 33.3%. During the same period, among 22 AIS patients with a history of CHD who were treated with PCSK9 inhibitors within 24 h of onset, 3 patients experienced END, accounting for 13.6%. Assuming the incidence of END in the intervention and control groups are 13.6% and 33.3%, respectively, with a two-sided α level of 0.05 and 80% power, we used the PASS software version 15 (NCSS, LCC, Taiwan 2017) to calculate the sample size. Each group requires 70 patients. Considering a 10% loss to follow-up, both groups will need 78 patients, making a total of at least 156 patients across both groups. The sample size calculation formula is as follows:

$$n=2({Z}_{\alpha /2}+{Z}_{\beta }{)}^{2}\cdot p(1-p)\div (\delta {)}^{2}$$

where:

*n* is the sample size per group.

*Z*_α_/2 is the critical value for a two-sided significance level (1.96 for *α* = 0.05).

Z_*β*_ is the critical value for power (0.84 for 80% power).

*p* is the estimated overall incidence rate.

*δ* is the expected difference between the two groups.

### Recruitment {15}

Potential participants will be recruited from the First Affiliated Hospital of Dalian Medical University. All eligible patients with AIS and a history of CHD within 24 h of onset will be invited to participate in this clinical trial. Recruitment will be managed by XG and XY and will span 18 months. Based on our preliminary intention-to-enroll survey, we estimate a recruitment rate of at least 80%. If we do not reach the required number of participants, we will extend the recruitment period as necessary. The study protocol will be thoroughly explained to potential participants by the investigators.

## Assignment of interventions: allocation

### Sequence generation {16a}

The allocation sequence for this study will be generated using computer-generated random numbers. The randomization will be stratified by gender. To reduce the predictability of the sequence, blocked randomization will be used to ensure a 1:1 ratio between the PI group (PCSK9 inhibitor with statin) and the AT group (statin monotherapy). Details of the block sizes and the randomization process will be documented separately and will remain unavailable to those who enroll participants or assign interventions.

### Concealment mechanism {16b}

The allocation sequence will be concealed using sequentially numbered, opaque, sealed envelopes. An independent researcher, not involved in recruitment, treatment, or assessment, will prepare these envelopes, each containing the group assignment (either the AT group or the PI group). After obtaining informed consent and completing baseline measurements, participants will be assigned the next sequential envelope by a designated researcher who is not involved in treatment or outcome assessment. The envelopes will remain sealed until the participant is assigned to an intervention, ensuring the allocation sequence is concealed from those enrolling participants and assigning interventions.

### Implementation {16c}

LP will generate the allocation sequence and prepare the opaque, sealed envelopes containing the group assignments. XG and XY will be responsible for enrolling participants and assigning them to their respective groups.

## Assignment of interventions: blinding

### Who will be blinded {17a}

Researchers responsible for data collection and outcome analysis will be blinded to group assignments until after data analysis is completed, as they will not be involved in participant assignment or intervention implementation.

### Procedure for unblinding if needed {17b}

Not applicable to study; the design is open label, so unblinding will not occur.

## Data collection and management

### Plans for assessment and collection of outcomes {18a}

The principal investigator will ensure that all study team members receive proper training, with access to written instructions and standard operating procedures. Regular meetings will facilitate communication and the sharing of new information. For quality assurance, the ethics committee (EKNZ) or an independent trial monitor may visit the research sites and will have direct access to source data and study-related files. All participant data will be kept strictly confidential and immediately uploaded to the designated cloud server after each assessment.

### Baseline

During the baseline assessment, demographic information will be gathered using standardized questionnaires. A comprehensive medical profile, including current and past treatments, allergies, personal background, surgical history, and family medical history, will be compiled. Assessments will include physical examinations and reliable laboratory tests (blood routine, urine routine, blood biochemistry, coagulation function, inflammatory factors). Additionally, electrocardiograms, NIHSS and mRS scores, documentation of concurrent medications, and recording of adverse events will be conducted.

### Treatment

Eligible patients will be randomly assigned to either the PI group (PCSK9 inhibitor combined with statin) or the AT group (statin monotherapy) to receive the designated interventions. On day 7 after enrollment, a physical examination and laboratory tests will be reassessed. The primary outcomes will be evaluated, mortality data collected, and concomitant medication usage documented.

### Follow-up

The follow-up period will start on day 8 after enrollment and continue until day 90, with evaluations on days 30 and 90. Assessments will include concomitant medications, adverse events, physical examinations, laboratory tests, NIHSS and mRS scores, and the occurrence of any recurrent cardiovascular or cerebrovascular events. If a participant experiences an endpoint event (recurrent cardiovascular or cerebrovascular event) during treatment or follow-up, the clinical study will be terminated, and active management will be initiated.

### NIHSS and mRS

The National Institutes of Health Stroke Scale (NIHSS) and the modified Rankin Scale (mRS) are standardized tools for evaluating stroke severity and outcomes. The NIHSS assesses various neurological functions such as consciousness, vision, motor function, sensation, speech, and language. Scores range from 0 to 42, with higher scores indicating more severe impairment [18]. Motor function is specifically assessed through Item 5 (Motor Arm), Item 6 (Motor Leg), and Item 7 (Limb Ataxia). The mRS evaluates disability and dependence in daily activities, ranging from 0 (no symptoms) to 6 (death). In this study, validated Chinese versions of both scales will be used [19]. Assessments will be conducted by two independent neurologists, and if discrepancies arise in their scores, a third senior neurologist will be consulted for the final assessment. All raters will receive comprehensive training before the trial begins, and repeated measurements will be conducted to ensure reliability and accuracy.

### Plans to promote participant retention and complete follow-up {18b}

To enhance participant retention and ensure complete follow-up, we will establish regular communication and engagement with participants, implement flexible scheduling for follow-up visits, and provide reminders through phone calls and messages.

### Data management {19}

This study will employ an electronic data management system (EDMS) with an electronic case report form (eCRF) constructed in accordance with the study protocol. Individuals responsible for data entry will be granted the necessary permissions to enter, modify, and resolve queries. Researchers will have permissions for modification, browsing, query resolution, and verification, whereas data management personnel will be permitted to browse, submit queries, and lock data.

To ensure the authenticity, completeness, and accuracy of the data, it is imperative that all trial records be fully completed with no empty or missing entries. Researchers are required to retain all original participant documents to maintain accurate and timely data. Original documents and medical records must be clear, detailed, and easily identifiable by clinical trial personnel. Data in the eCRF can only be modified by authorized researchers. All original trial data records will be preserved for a minimum of 5 years.

### Confidentiality {27}

Personal information about participants will be collected, shared, and maintained with strict confidentiality throughout the trial. Each participant will be assigned a unique identification number, which will label all study-related documents and data to ensure anonymity. Data will be stored in secure, password-protected electronic databases, and physical records will be kept in locked cabinets accessible only to authorized personnel.

Data sharing will be restricted to the research team and authorized monitors, auditors, and regulatory authorities as required. Any shared data will be de-identified. Electronic data transfers will be encrypted for added security.

Regular audits and data checks will ensure compliance with confidentiality protocols. After the trial, personal data will be securely stored for at least 5 years. Participants’ personal information will not be disclosed in any publications or reports.

### Plans for collection, laboratory evaluation, and storage of biological specimens for genetic or molecular analysis in this trial/future use {33}

Not applicable. This study does not involve the use of biological samples for genetic or molecular analysis.

## Statistical methods

### Statistical methods for primary and secondary outcomes {20a}

Once all participants have completed their assessments, data analysis will commence. To reduce bias, the data analyst will remain blinded during the analysis process. Only participants who strictly adhere to the intervention protocol will be included in the analysis. Data that are missing or deviate from the protocol will be excluded from the analysis.

The primary and secondary outcomes will be combined as dependent variables in the generalized linear model (GLM). First, a univariate analysis of the treatment groups will be performed to determine the unadjusted risk ratio (RR) and odds ratio (OR), along with a 95% confidence interval (CI). Next, continuous variables like the NIHSS score at admission, mRS score, and age will serve as covariates. Categorical variables such as sex, hypertension, diabetes, and prior strokes will be included as factors in the GLM to reduce variance error. If the outcomes show statistical significance for the main effects, interaction effects will be used to assess differences between the groups. Changes in blood biochemical indices will be analyzed using repeated measures ANOVA or mixed-effects models, accounting for measurements taken at baseline, 7 days, 30 days, and 90 days. Changes in inflammatory factors (IL-6, IL-8, TNF-α) from baseline to 7 days will be evaluated using paired *t*-tests or Wilcoxon signed-rank tests, based on data distribution. Sensitivity analysis will use the criterion for severe END, defined as either a 4-point increase in NIHSS or a 2-point worsening in motor function based on a higher threshold. All analyses will be conducted using SPSS (IBM Corp., Armonk, NY, USA), with a significance threshold of *α* = 0.05.

### Interim analyses {21b}

Not applicable to the study. Data analysis will not commence until all participants have completed the study.

### Methods for additional analyses (e.g., subgroup analyses) {20b}

Additional analyses will explore potential subgroup differences and interaction effects. Subgroup analyses will stratify by age, gender, baseline stroke severity (NIHSS), and comorbid conditions such as diabetes or hypertension. These analyses will be conducted using stratified chi-square tests for categorical outcomes and stratified Wilcoxon rank-sum tests or mixed-effects models for continuous outcomes. Interaction effects will be assessed with multivariable logistic regression models for binary outcomes. All statistical tests will be two-tailed, with a *p*-value of less than 0.05 considered statistically significant.

### Methods in analysis to handle protocol non-adherence and any statistical methods to handle missing data {20c}

To address protocol non-adherence, we will define the analysis populations as follows: the intention-to-treat (ITT) population will include all randomized participants who have at least one treatment record and will be used for analyzing the primary outcome. The per-protocol (PP) population will include only those participants who fully adhere to the study protocol without major deviations and will be used for analyzing the secondary outcomes. Experience indicates that dropouts are rare. If the proportion of missing data exceeds 10%, multiple imputation techniques will be employed to ensure the robustness of our study results.

### Plans to give access to the full protocol, participant-level data, and statistical code {31c}

Upon reasonable request, the full protocol, participant-level data, and statistical code will be made available from the corresponding author.

## Oversight and monitoring

### Composition of the coordinating center and trial steering committee {5d}

The Department of Neurology at the First Affiliated Hospital of Dalian Medical University will serve as the trial coordinating center. The trial steering committee (TSC) will consist of one principal investigator and two co-investigators. The TSC will be responsible for ensuring the trial is conducted according to the protocol and for providing oversight throughout the study. This will include approving the final protocol, monitoring study progress, making decisions regarding any protocol amendments, and ensuring the overall integrity of the trial. The committee will meet bi-monthly to review progress and address any issues that may arise.

The trial management committee (TMC) will consist of two co-investigators, two master’s students, and an independent scientific assistant. The TMC will be responsible for the day-to-day management of the trial, including planning, coordination, and execution of the study. Their duties will encompass overseeing patient recruitment, ensuring protocol compliance, managing data collection and quality control, and maintaining participant safety and trial integrity. The independent scientific assistant will be solely responsible for generating the random allocation sequence, ensuring unbiased assignment of participants. The TMC will hold weekly meetings to review trial operations and address any immediate concerns.

### Composition of the data monitoring committee, its role and reporting structure {21a}

In consideration of the relatively low-risk profile of the trial as determined by the hospital’s ethics committee and the study’s steering committee, there will be no formal data monitoring committee (DMC). Instead, an internal trial monitor, independent of the study team, will be responsible for regular onsite monitoring according to the established monitoring plan.

### Adverse event reporting and harms {22}

Adverse events (AEs) are defined as any clinical manifestation, syndrome, or worsening of pre-existing conditions that affect the health of participants during the clinical trial. These events may not necessarily be related to the PCSK9 inhibitor and statin treatment and can include new illnesses or exacerbation of existing symptoms. All solicited and spontaneously reported AEs, as well as other unintended effects of trial interventions or conduct, will be documented from participant enrollment until the study concludes. All AEs will be recorded in the electronic case report form (eCRF). Participants will be monitored for AEs through regular visits, phone calls, and during any unscheduled visits. The study team will assess AEs for severity, duration, and causality. The severity of AEs will be classified according to the Common Terminology Criteria for Adverse Events (CTCAE), and causality will be determined based on the relationship between the AE and the study intervention, categorized as unrelated, unlikely, possible, probable, or definite. Serious adverse events (SAEs) will be reported to the ethics committee and relevant regulatory authorities within 24 h of awareness by researchers. Following the occurrence of an SAE, researchers must provide appropriate treatment to the participant and submit a follow-up report to the ethics committee within 15 days, detailing the cause of the event and the measures taken.

### Frequency and plans for auditing trial conduct {23}

The hospital’s ethics committee will audit the study every 6 months to ensure compliance with the protocol, ethical guidelines, and regulatory requirements. The auditing process will include a comprehensive review of trial documentation, participant records, and data management practices.

### Plans for communicating important protocol amendments to relevant parties (e.g., trial participants, ethical committees) {25}

Any modifications will first be discussed within the Steering Committee to reach a consensus. Amendments will then be submitted to the hospital’s Ethics Committee and the Department of Scientific Research for approval. Once approved, a formal document outlining the protocol revisions will be provided to all relevant parties. Trial participants will be informed of any changes that may affect their participation, and their consent will be re-obtained if necessary.

### Dissemination plans {31a}

At the conclusion of the study, participants interested in the results will receive a summary via email. The study findings will be published in peer-reviewed scientific journals and presented to the medical community through talks at national and international conferences and seminars. Furthermore, the results will be made available in the Chinese Clinical Trial Registry and relevant databases to ensure open access to the data.

## Discussion

This study aims to evaluate the efficacy and safety of combining PCSK9 inhibitors with statins compared to statin monotherapy in preventing early neurological deterioration (END) in acute ischemic stroke (AIS) patients with a history of coronary heart disease (CHD). Our goal is to develop a better treatment approach for these patients, which will reduce the occurrence of END and enhance their overall prognosis.

This study is a single-center, randomized, parallel-group trial that ensures comparability between the intervention and control groups after randomization. The inclusion criteria are clearly defined to select a relevant patient population for the research. Changes in NIHSS scores will be used to assess END. Meanwhile, mRS scores will evaluate disease prognosis, offering an objective reflection of the patients’ neurological status.

**Fig. 3:**
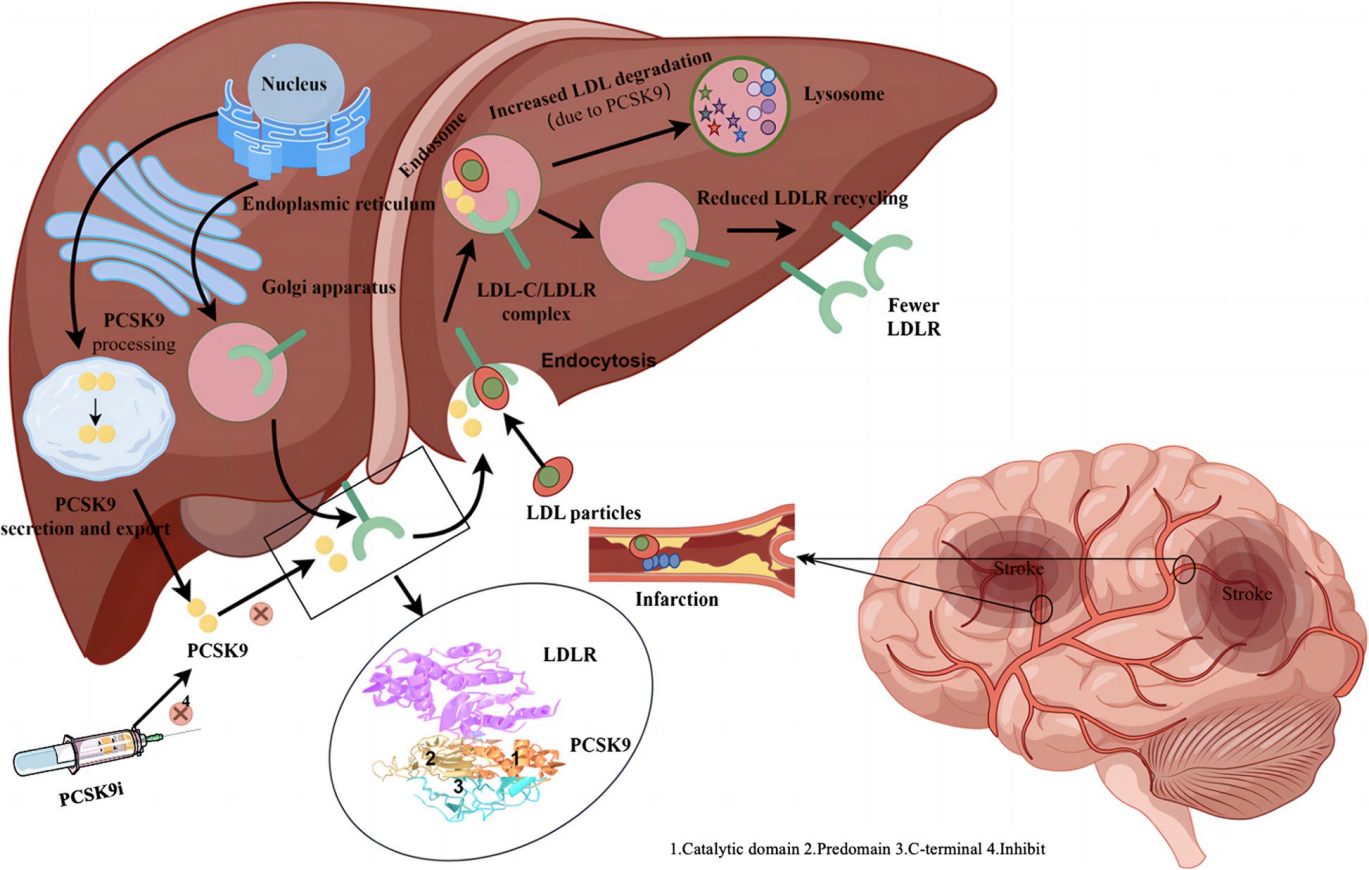
Potential mechanisms of action of PCSK9 Inhibitors

PCSK9 inhibitors (mechanism of action in Fig. 3) are effective at lowering LDL cholesterol levels and reducing cardiovascular risk [20]. However, their role in stroke, particularly in preventing END, has not been thoroughly investigated. Our preliminary trial results indicate that the combination of evolocumab and statins administered within 24 h after AIS effectively reduces the incidence of END. This combination also improves clinical outcomes at 90 days. Additionally, this combination therapy inhibits the increase of IL-6 levels 7 days after the stroke [21]. Building on this foundation, we are focusing on AIS patients with a history of CHD, who have a higher incidence of END. This study aims to compare the incidence of END between two lipid-lowering treatment groups to evaluate the preventive efficacy of evolocumab in this patient population. Furthermore, this research will validate the efficacy of PCSK9 inhibitors in reducing LDL-C levels while further exploring their role in mitigating inflammatory responses.

However, this study has its limitations. Currently, there are no reported incidence rates of END in AIS patients with a history of CHD. Therefore, we can only estimate the occurrence using a small sample size from prior clinical observations, leading to a limited number of cases. The single-center design and relatively small sample size may limit the generalizability of its results. Additionally, the relatively short follow-up period may not capture long-term outcomes. Future multi-center studies with larger sample sizes and longer follow-up periods are necessary to validate our results and explore the long-term benefits and risks of combined PCSK9 inhibitors and statin therapy in stroke patients.

### Trial status

This trial is conducted under protocol version 1.1, dated November 30, 2023. Recruitment commenced in December 2023 and is anticipated to be completed by November 2025.

## Supplementary Information

Supplementary Material 1.

## Data Availability

Data from this study are available upon reasonable request from the corresponding authors. The data are monitored by the corresponding authors and the scientific research department of the hospital.

## Abbreviations

ECG: Electrocardiogram
CT: Computed tomography
MRI: Magnetic resonance imaging
END: Early neurological deterioration
CHD: Coronary heart disease
NIHSS: National Institutes of Health Stroke Scale
mRS: Modified Rankin Scale
IL-6: Interleukin-6
IL-8: Interleukin-8
TNF-α: Tumor necrosis factor-alpha
