# Supplementary material for: Effect of PCSK9 inhibitor on early neurological deterioration in acute ischemic stroke patients with a history of coronary heart disease: a study protocol for a randomized controlled trial in Dalian, China
Source: Trials. 2025 Jan 6;26:7. doi: 10.1186/s13063-024-08709-2 (PMC11702183; doi:10.1186/s13063-024-08709-2)
Supplement: Supplementary file 1 — Supplementary Material 1. [file 13063_2024_8709_MOESM1_ESM.pdf]

·临床研究·

中文版美国国立卫生院脑卒中量表的信度与效度研究

侯东哲 张颖 巫嘉陵 李毅 安中平

【摘 要】 **目的** 研究脑卒中患者中文版美国国立卫生院脑卒中量表(NIHSS)的信度和效度。**方法** 选取 156 例急性脑卒中患者分别由不同评定者于入院当天、入院第 15 天、发病后第 90 天对同一脑卒中患者进行 NIHSS 的评定。用 Kappa 值表示重测信度与评定者间信度,用 Cronbach α 值表示分半信度、内部一致性信度。采用 Spearman 相关分析预测效度,采用因子分析方法评定结构的效度。**结果** 所有重测信度、评定者间信度的 Kappa 值均有统计学意义($P<0.01$);分半信度:NIHSS 奇偶项两部分的 Cronbach α 值分别为 0.809、0.857,两部分的 Spearman 相关系数为 0.873;内部一致信度:Cronbach α 值为 0.796;效度研究中,KMO 统计量为 0.769,Bartlett 球形检验拒绝相关阵是单位阵($P<0.01$);入院当天及发病第 15 天的 NIHSS 评分与发病第 90 天的 NIHSS 评分呈正相关($P<0.01$)。**结论** NIHSS 中文版作为评定神经功能缺损的量表,其重测信度和评定者间信度均较佳,内部一致性好;具有结构效度、预测效度,可应用于脑卒中的评定。

【关键词】 脑卒中; 量表; 信度; 效度

脑卒中的发病率和病死率高,致残率亦高,严重威胁着人类的身体健康[1]。CT、MRI 及脑血管成像等技术可显示脑卒中病灶的部位、大小及性质,脑卒中量表是显示功能变化不可缺少的手段,可用来判断病情变化、评估治疗效果、预测脑卒中的结局[2]。现国内外脑卒中量表很多,美国国立卫生院脑卒中量表(National Institutes of Health stroke scale, NIHSS)[3]在国内外应用比较广泛,本研究旨在探讨中文版 NIHSS 的信度和效度,并分析其在患病初期的评定值与疾病预后的相关性。

资料与方法

一、研究对象

纳入标准:符合 1995 年第四届全国脑血管病学术会议制定的脑卒中诊断标准[4],并经头部 CT 和/或 MRI 检查证实;发病时间 <48 h。

排除标准:发病前有脑卒中并使或存在神经功能缺失者;病灶不明确;蛛网膜下腔出血;短暂性脑缺血发作(transient ischemic attack, TIA);精神疾患或不能合作者;有严重心、肝、肾等严重并发症。

选取 2009 年 11 月至 2011 年 4 月天津市环湖医院收治的且符合上述标准的急性脑卒中患者 156 例,其中男 96 例,女 60 例;年龄 42 ~ 76 岁,平均(58.43 ± 17.35)岁;脑梗死 121 例,脑出血 35 例。而在 121 例脑梗死患者中,前循环梗死 82 例,后循环梗死 39 例。

二、评定和评价方法

量表评定:由 2 位医师分别对同一脑卒中患者于入院当天、入院第 15 天及入院第 90 天用中文版 NIHSS 进行评定,评定后 2 h,由该医生再次进行上述评定。所有评定者在本研究开始前均接受 NIHSS 中文版的评定培训。

评价指标:信度分析,包括重测信度、评定者间信度,应用分半信度及内部一致性信度;效度分析包括结构效度、同时效度和预测效度[5]。

三、统计学分析

采用 SPSS 13.0 版软件进行统计学分析,检测数据以($\bar{x} \pm s$)表示,样本间均数的比较应用 F 检验,重测信度与评定者间信度用 Kappa 值表示,分半信度、内部一致性信度用 Cronbach α值表示。同时效度与预测效度采用 Spearman 相关分析。结构效度采用 KMO 检验和 Bartlett 球形检验、因子分析的方法分析。$P<0.05$ 为差异有统计学意义。

结 果

一、NIHSS 评定值

脑卒中患者入院当天、入院第 15 天及发病后第 90 天分别由 2 位医生相隔 2 h(2 次)进行中文版 NIHSS 评定,NIHSS 评定值之间差异无统计学意义($P>0.05$),详见表 1。

表 1 不同评定时间点 2 位医生 2 次的 NIHSS 评定值比较(分,$\bar{x} \pm s$)

评定时间点	医师 A		医师 B	
	第一次	第二次	第一次	第二次
入院当天	8.27 ± 5.02	8.32 ± 5.19	8.26 ± 5.07	8.33 ± 4.36
入院第 15 天	5.36 ± 3.21	5.51 ± 3.38	5.41 ± 3.02	5.52 ± 3.17
发病后第 90 天	3.27 ± 2.08	3.35 ± 2.31	3.29 ± 1.98	3.34 ± 1.78

二、信度分析

所有重测信度、评定者间信度的 Kappa 值均有统计学意义($P<0.01$),详见表 2;分半信度:中文版 NIHSS 奇偶项两部分的 Cronbach α 值分别为 0.809 和 0.857,两部分的 Spearman 相关系数为 0.873;内部一致信度:Cronbach α 值为 0.796。

三、效度分析

1. 结构效度:本研究中患者的 KMO 统计量为 0.769,Bartlett 球形检验拒绝相关阵是单位阵($P<0.01$)。采用主成份因子分析法,取特征根大于 1 的因子,得到公因子,其累计贡献率为 0.6252,面瘫在 4 个公因子中的负荷均未 >0.4,意识指令、意识

DOI:10.3760/cma.j.issn.0254-1424.2012.05.013

作者单位:300121 天津,天津市环湖医院内科(侯东哲、巫嘉陵、李毅、安中平);南开大学人民医院康复医学科(张颖)

通信作者:张颖,Email: ying_zhang_graduate@yahoo.com

提问、意识水平、右上肢肌力、右下肢肌力、语言功能负荷于因子 1；水平凝视、左上肢肌力、左下肢肌力负荷于因子 2；共济失调、构音障碍负荷于因子 3；视野、感觉、偏侧忽视负荷于因子 4。第一公因子的特征值为 5.934，随着共因子数目的增加，其特征值降低。当公因子的数目接近条目数(15)时，其特征值接近为 0(图 1)。

表 2　NIHSS 的信度评定(Kappa 值)

评分内容	重测信度	评定者间信度
意识水平	0.680	0.651
意识提问	0.698	0.703
意识指令	0.799	0.729
水平凝视功能	0.788	0.772
视野	0.761	0.711
面瘫	0.628	0.679
左上肢运动	0.865	0.821
右上肢运动	0.869	0.809
左下肢运动	0.912	0.856
右下肢运动	0.869	0.824
共济失调	0.484	0.442
语言	0.709	0.642
构音障碍	0.659	0.617
感觉	0.711	0.731
忽视	0.677	0.654

注：$P<0.01$

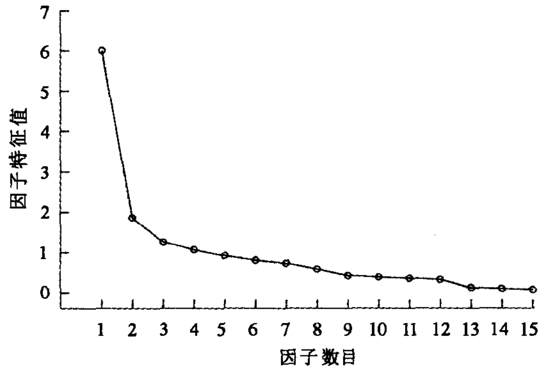

图 1　NIHSS 因子分析散点图

2. 预测效度：入院当天、发病第 15 天的中文版 NIHSS 评分与发病后第 90 天的中文版 NIHSS 评分结果呈正相关，r 值分别为 0.569 和 0.656，差异有统计学意义($P<0.01$)。

讨　论

近年来美国国立卫生研究院脑卒中量表[6]在临床的应用逐渐增多，用于病情的评定，康复计划的制订和效果检测及患者预后的预测[3,7]。其包括 15 项条目[6,8]：意识情况、眼外肌运动、视野、面肌功能、肢体运动、感觉、共济失调、语言功能、构音障碍和偏侧忽略等项目，其中肢体远端运动功能的检测是附加项目。NIHSS 英文版[6,9]已被证明有很好的信度和效度。量表从一种文字转换为另一种文字并不是简单的翻译过程，需做严格的信度和效度分析，本研究即对中文版 NIHSS 的信度和效度进行分析，且分析其患病初期评定值与疾病预后的相关性。

信度主要评价量表的精确性、稳定性和可靠性，Kappa 值是最好的评测指标[10-11]。NIHSS 英文版已经在前瞻性临床试验中已经证实有很好的信度与效度。本研究中重测信度及评定者间信度 4 项信度很好，10 项较好，1 项中等。其中共济失调的信度中等，本组完全前循环梗死患者较多，患者认知能力较差的比例较大，可能会影响评定结果。评定者间信度较高的项目为肌力评定，这是医务工作者临床常用项目，熟练程度高，故信度较高。本研究显示中文版 NIHSS 的分半信度，内部一致性信度均较高，但较国外学者[12]的报道低，需进一步加强对评定者的培训，以提高中文版 NIHSS 评定信度。

效度分析是检验测定中的系统误差，主要评价量表的准确度、有效性和正确性。本研究全部患者中文版 NIHSS 评分结果显示 NIHSS 有很好的结构效度，有 4 个公因子，意识指令、意识提问、意识水平、右上肢肌力、右下肢肌力、语言功能，负荷于因子 1，反映左侧大脑皮质和运动功能；水平凝视、左上肢肌力、左下肢肌力，负荷于因子 2，反映右侧大脑运动功能；共济失调、构音障碍，负荷于因子 3，反映脑干、小脑的功能变化；视野、感觉、偏侧忽视，负荷于因子 4，可能反映的是右侧大脑皮质功能情况，感觉和视野受损在右侧半球皮质功能障碍时表现突出，可能与左侧半球损害时，患者有严重意识障碍或失语，不能描述感觉或视野的障碍有关。中文版 NIHSS 在前瞻性临床试验中已经证实对脑卒中的远期结局亦有预测效度[12]。

综上所述，中文版 NIHSS 是评定神经功能缺损的量表，重测信度和评定者间信度佳，内部一致性好；具有结构效度、预测效度，可应用于脑卒中的评定。

参　考　文　献

[1] 赵彦超. 脑卒中的预防干预措施分析. 中国实用神经疾病杂志，2011，14：52-53.

[2] Rimer JH. Use of the ICF in identifying factors that impact participation in physical activity/rehabilitation among people with disabilities. Disabil Rehabil，2006，28：1087-1095.

[3] Brott TG，Adams HP Jr，Olinger CP，et al. Measurements of acute cerebral infarction：a clinical examination scale. Stroke，1989，20：864-870.

[4] 中华神经科学会，中华神经外科学会. 各类脑血管疾病诊断要点. 中华神经科杂志，1996，29：379-380.

[5] Goldstein LB，Samsa GP. Reliability of the National Institutes of Health stroke scale：extension to non-neurologists in the context of a clinical trial. Stroke，1997，28：307-310.

[6] Tseng MC，Chang KC. Stroke severity and early recovery after first-ever ischemic stroke：results of a hospital-based study in Taiwan. Health Policy，2006，79：73-78.

[7] Schlegel DJ，Tanne D，Demchuk AM，et al. Prediction of hospital disposition after thrombolysis for acute ischemic stroke using the National Institutes of Health Stroke Scale. Arch Neurol，2004，61：1061-1064.

[8] Tei H，Uchiyama S，Usui T. Clinical-diffusion mismatch defined by NIHSS and ASPECTS in non-lacunar anterior circulation infarction. J Neurol，2007，254：340-346.

[9] Lyden P，Raman R，Liu L，et al. NIHSS training and certification using a new digital video disk is reliable. Stroke，2005，36：2446-2449.

[10] Posner KL，Sampson PD，Caplan RA，et al. Measuring interrater reliability among multiple raters：an example of methods for nominal data.

Stat Med, 1990, 9: 1103-1115.
[11] Cyr L, Francis K. Measures of clinical agreement for nominal and categorical data: the kappa coefficient. Comput Biol Med, 1992, 22: 239-246.
[12] Bruno A, Saha C, Williams LS. Using change in the National Institutes of Health Stroke Scale to measure treatment effect in acute stroke trials. Stroke, 2006, 37: 920-921.

(修回日期:2012-04-16)
(本文编辑:汪　玲)

规范三级康复治疗对脑卒中后偏瘫伴肩痛患者上肢运动功能及其生活质量的影响

张继荣　吴霜　黄宇　郑栋华　裴强　龙燕　冯丽丽　陈景舟　石承龙

【摘　要】 目的　探讨规范三级康复治疗对脑卒中后偏瘫伴肩痛患者的上肢运动功能、生活质量以及疼痛程度的影响。**方法**　脑卒中后偏瘫伴肩痛患者62例,按随机数字表法分为康复组和对照组,每组患者31例,2组患者均采用相同的常规康复治疗,康复组增加规范的三级康复治疗。2组患者均于治疗前和治疗6个月后(治疗后)分别采用简式Fugle-Meyer运动功能评定量表(FMA)、世界卫生组织生活质量评定简表(WHOQOL)和目测类比法(VAS)评定其上肢运动功能、生活质量及肩痛程度。**结果**　治疗后,康复组患者各项评分与组内治疗前和对照组治疗后比较,差异均有统计学意义($P<0.05$);对照组患者各项评分与组内治疗前比较,差异均无统计学意义($P>0.05$)。治疗后,治疗组肩痛例数降为16例(51.6%),与对照组的22例(71.0%)比较,差异有统计学意义($P<0.05$)。**结论**　规范的三级康复治疗可显著改善脑卒中后偏瘫伴肩痛患者的上肢运动功能、生活质量和疼痛程度。

【关键词】　三级康复;　偏瘫肩痛;　上肢运动功能;　生活质量

肩痛是脑卒中后偏瘫患者临床常见的并发症之一,多发于脑卒中后2周内[1]或1~3个月[2],严重影响患者的上肢运动功能及其生活质量。大量研究发现,早期规范的康复治疗可有效地预防或治疗脑卒中后偏瘫患者的肩痛[3-5],促进患者上肢运动功能的恢复,提高生活质量[6]。2010年12月至2011年12月,我院康复科通过对脑卒中后偏瘫伴肩痛患者实施早期规范的三级康复治疗[4-5],获得了显著疗效。报道如下。

资料与方法

一、一般资料

入选标准:①符合第四届脑血管病会议通过的脑血管病诊断标准[7],并经头颅CT或MRI确诊的初次发病患者;②病程≤3周,生命体征稳定48 h,神志基本恢复(格拉斯哥昏迷量表评分>8分);③年龄40~80岁;④有一侧肢体功能障碍并伴有肩痛者;⑤均签署知情同意书。排除标准:严重的心、肺、肝、肾等脏器疾病或器官衰竭的患者;恶性肿瘤、四肢瘫、聋哑或有痴呆、精神病的患者以及不能随访的患者。

选取2010年12月至2011年12月本院收治的符合上述标准的脑卒中后偏瘫伴肩痛患者62例,采用随机数字表法分为康复组和对照组,每组患者31例,2组患者一般资料比较,差异无统计学意义($P>0.05$),具有可比性,详见表1。

二、治疗方法

2组患者急性期均接受相同的常规康复治疗,康复组在常规康复治疗的基础实施规范三级康复治疗。

(一)常规康复治疗

常规康复治疗包括中频电、磁疗、超短波或压力治疗等物理因子治疗。

1. 中频电疗法:采用北京产多功能电脑中频仪(ECM99-ⅡB),频率为2~8 kHz,输出电流0~100 mA,调制频率0~150 Hz,将电极置于患者偏瘫肢体的上肢伸肌群及下肢屈肌群,输出处方为功能性电刺激,刺激强度为耐受限,每日1次,每次20 min,10 d为1个疗程,连续3个疗程。

2. 磁疗:采用日本产温热磁场治疗仪(TM3200),将20 cm×15 cm的长方形电极置于患肩,中温(约50 ℃)剂量输出,每日1次,每次20 min,10 d为1个疗程,连续3个疗程。

3. 超短波疗法:采用汕头产立式超短波治疗仪(DC-C-BⅡ),频率为40.68 MHz,最大输出功率为200 W,将2个20 cm×15 cm的长方形电极于患肩对置,微热量输出,每日1次,每次15 min,10 d为1个疗程,连续3个疗程。

4. 压力疗法:采用韩国产四肢循环治疗仪(DL2003V6),压力范围20~200 mmHg(1 mmHg=0.133 kPa),输出功率26 W,将气囊套于患侧上下肢体,中档压力输出,每日1次,每次18 min,10 d为1个疗程,连续3个疗程。

(二)规范三级康复治疗

规范三级康复治疗遵循参考文献[8]实施。

1. 一级康复:入组后第1个月(早期),患者在病房行早期康复治疗,包括抗痉挛体位摆放、患肢被动活动(肩胛带运动时应注意避免过度牵拉上肢)、桥式训练、神经肌肉促通技术应用、从卧位到坐位再到站位的转移训练及坐位平衡训练、床上基本日常生活活动能力训练等。治疗同时指导家属或陪护正确的护理及辅助训练的方法,减少误用和废用综合征。每日治疗1次,每次45 min,每周治疗5 d。

DOI:10.3760/cma.j.issn.0254-1424.2012.05.014
作者单位:550004 贵阳,贵州省贵阳医学院附属医院康复科
通信作者:吴霜,Email: wus212@sina.com
